# Autofluorescence Detection of Bronchial Tumors With the D-Light/AF

**DOI:** 10.1155/DTE.5.105

**Published:** 1999

**Authors:** K. Häußinger, F. Stanzel, R. M. Huber, J. Pichler, H. Stepp

**Affiliations:** 1 Clinic for Pneumology Fachklinik München – Gauting Robert-Koch-Allee 2 Gauting D-82131 Germany; 2 Division of Pneumology Department of Medicine Klinikum Innenstadt University of Munich Germany; 3 Laser Research Institute Department of Urology University of Munich Germany

## Abstract

We present a newly developed diagnostic system combining a conventional light source (white light mode and two different fluorescence excitation modes), a bronchoscope and optionally a highly sensitive camera (Baumgartner *et al., Photochem. Photobiol.* 1987; 46(5): 759–763). Routine diagnostics can be performed with the autofluorescence bronchoscopy (AFB) and the white light bronchoscopy (WLB) in one diagnostic procedure. The image is visible directly with the naked eye. The system was evaluated in a pilot study including 60 patients. Two hundred and sixty-four biopsies were taken to detect premalignant and malignant findings (Stanzel *et al.,* Contribution to 10th World Congress for Bronchology, June 1998). The sensitivity of the combination of WLB and AFB was 2.8 times higher than that of the conventional WLB. The specificity decreased from 94% (WLB) to 89% (WLB + AFB). The results of this preliminary pilot study are being confirmed in a multicenter study, which will begin at seven European centers.


*Diagnostic and Therapeutic Endoscopy*, Vol. 5, pp. 105–112
Reprints available directly from the publisher
Photocopying permitted by license only

© 1999 OPA (Overseas Publishers Association) N.V.
Published by license under
the Harwood Academic Publishers imprint,
part of The Gordon and Breach Publishing Group.
Printed in Singapore.

### Autofluorescence Detection of Bronchial Tumors with the D-Light/AF

K. HÄUßINGER[a,*], F. STANZEL[a], R.M. HUBER[b], J. PICHLER[c] and H. STEPP[c]

[a]*Clinic for Pneumology, Fachklinik München – Gauting, Robert-Koch-Allee 2, D-82131 Gauting, Germany;* [b]*Division of Pneumology, Department of Medicine, Klinikum Innenstadt, University of Munich, Germany;* [c]*Laser Research Institute, Department of Urology, University of Munich, Germany*

**We present a newly developed diagnostic system combining a conventional light source (white light mode and two different fluorescence excitation modes), a bronchoscope and optionally a highly sensitive camera (Baumgartner *et al.*, *Photochem. Photobiol.* 1987; 46(5): 759–763). Routine diagnostics can be performed with the autofluorescence bronchoscopy (AFB) and the white light bronchoscopy (WLB) in one diagnostic procedure. The image is visible directly with the naked eye. The system was evaluated in a pilot study including 60 patients. Two hundred and sixty-four biopsies were taken to detect premalignant and malignant findings (Stanzel *et al.*, Contribution to 10th World Congress for Bronchology, June 1998). The sensitivity of the combination of WLB and AFB was 2.8 times higher than that of the conventional WLB. The specificity decreased from 94% (WLB) to 89% (WLB + AFB). The results of this preliminary pilot study are being confirmed in a multicenter study, which will begin at seven European centers.**

*Keywords:* Autofluorescence, Bronchoscopy, D-Light AF, Early detection, Lung cancer, CIS, Dysplasia

### INTRODUCTION

Invasive bronchogenic lung cancer represents the final stage of a long sequence of cellular and tissue alterations [3]. Animal experiments have shown hyperplasia and metaplasia of the bronchial mucosa to be a gradual development up to dysplasia, carcinoma *in situ* (CIS) and invasive carcinoma. Premalignancies are clearly defined by histomorphological criteria by WHO: Dysplasia is considered as premalignancy and is characterized by cellular atypia in metaplastic epithelial tissue. Carcinoma *in situ* is defined by nuclear and cellular atypia in partially abolished and irregular cell layers. In bronchogenic carcinoma the basement membrane is destroyed and the stroma is infiltrated.

Endoscopy for detection of early lung cancer may be helpful for two reasons: Firstly carcinogenesis is a

* Corresponding author. Tel.: +49 89 85791 7301. Fax: +49 89 85791 7305.

slow process evolving over years, and is estimated to take 3–4 years for dysplasia and approximately a further six months for CIS [4,5]. Secondly about 50–60% of all lung cancers, especially squamous cell carcinomas, develop in the central airways. These cancers can be detected by endoscopy, but are generally roentgenographically occult [6].

The main problem for conventional bronchoscopy is its low sensitivity and specificity for detection of early malignant changes [7]. Premalignant lesions usually are small and only some cell layers thick [8]. Their endoscopic signs therefore are very subtle and can be missed even by experienced bronchoscopists. To improve the sensitivity and specificity of conventional endoscopy, diagnostic fluorescence procedures were developed [9]. The principles of these procedures are based on a difference in fluorescence between normal tissue and premalignant and malignant tissue. This difference can be visualized by introducing stimulating light of a special wavelength (380–460 nm) [10].

Two basic methods are available:

- the detection of the specific autofluorescence of tumor tissue and normal tissue [11],
- the application of photosensitive drugs, which selectively accumulate in tumor tissue and cause a specific drug induced fluorescence [12] (contribution by Dr. Huber *et al.*).

In the following we will describe the detection of the specific autofluorescence by a special technical system, developed by the Karl Storz company (Tuttlingen, Germany) in collaboration with the Laserforschüngslabor (LFL) of the University of Munich, Germany.

### MATERIALS AND METHODS

### Technical Development, Final Equipment

First attempts with the fluorescence detection were accomplished with hematoporphyrin derivatives as marker substance and a krypton laser for excitation [13]. Hematoporphyrin derivatives later on were replaced by 5-aminolevulinic acid (Medac company, Hamburg) [14], the krypton laser was replaced by a conventional xenon light source emitting a broadbanded excitation light (D-Light, Karl Storz company, Germany) [15]. A detection filter on the ocular of the bronchoscope allowed observation of the flourescence light with the naked eye. With this method autofluorescence of low intensity could be detected even in patients without prior application of 5-aminolevulinic acid [16]. Both the autofluorescence and the contrast between tumor and normal tissue were very low. To increase the contrast and the light intensity of the D-Light system, the detection filters and the light guidance devices of the bronchoscope were optimized. The detection filters were modified allowing only the passage of a small part of the blue excitation light. After modification we obtained a higher color contrast, a higher light intensity and a more 3-D-image. Finally the application of photosensitive drugs became unnecessary.

The system in the actual version can be used with flexible as well as with rigid bronchoscopes. The findings are documented with a highly sensitive integrating camera which is put directly on the ocular of the bronchoscope. The technical details of the present system are explained in the contribution by Dr. M. Leonhard. Optionally and experimentally spectral detection of the specific autoflourescence by a beam splitter can be added [17]. Figure 1 shows

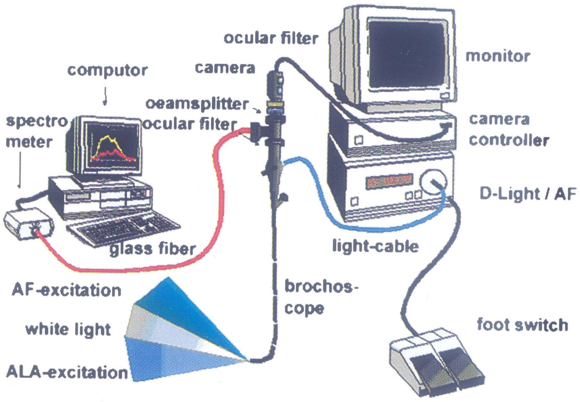

FIGURE 1 Technical setup.

the complete system D-Light/AF with bronchoscope and camera as well as the optional spectrometer.

The color of the normal mucosa is dominated by a green fluorescence, while malignant tissues are indicated by changes in the color, relief and fine structure of the mucosal surface.

### Clinical Study

The system D-Light/AF was tested in a pilot study including 60 patients (44 males, 16 females) with increased risk for developing bronchial carcinoma. Overall 264 biopsies were taken. According to the criteria of the International Association of Study of Lung Cancer (IASLC) [18] patients who met the following criteria were included:

- Radiological or clinical suspicion of carcinoma;
- Postoperative care (resected bronchial carcinoma);
- Positive cytological findings;
- Previous positive findings of dysplasia/CIS;
- Known bronchial carcinoma (e.g. staging);
- Smoker older than 40 years and evidence of COPD and/or occupational exposure.

Bronchoscopy was performed with local anesthesia with flexible instruments or with general anesthesia with rigid tubes in combination with flexible bronchoscopes. The detection of autofluorescence was performed only by flexible bronchoscopes. In some individual cases autofluorescence detection in the trachea and the main stem bronchii was performed by rigid optical devices to obtain a better documentation.

The investigation first started with white light mode switching over to blue light mode for the detection of the specific autofluorescence. Biopsies were taken from all suspicious areas detected either by WLB and/or AFB. Additionally in every patient two random biopsies were taken from normal bronchial tissue. The biopsies were described in WFB as well as in AFB by the following classification:

1. normal appearance, unsuspicious;
2. non-specific changes:
   - e.g. scars, granulomas, swelling, anatomical anomalies,
   - inflammation (acute chronic bronchitis), confessed (preceeding) location of biopsies;
3. suspicion of malignant changes;
4. visible tumor (e.g. exophyt).

The mean age of the patients investigated was 62.2 years (37–80 years). Forty-nine patients underwent bronchoscopy because of radiological or clinical suspicion for malignancy, 4 patients had positive findings in cytology, 11 patients had undergone surgical treatment due to bronchial carcinoma.

### RESULTS

Mean time for overall bronchoscopy was 25 min on average. Additional AFB lasted 7 min on average. For the detection rate of dysplasia and carcinoma *in situ* we found a clear increase of sensitivity and for normal tissue a minor decrease of specificity: We found 5 cases of mild dysplasia (1 in WLB, 3 in AFB, 1 in WLB + AFB), 6 cases of moderate to severe dysplasia (3 in AFB, 1 in WLB, 2 non-specific in WLB + AFB), 1 case of carcinoma *in situ* (suspicious in WLB + AFB) and 36 tumors (1 in WLB, 1 in AFB, 28 in AFB + WLB, 6 non-specific in WLB and AFB). The prevalence for moderate dysplasia, severe dysplasia and CIS was 7% for WLB alone and 12% for WLB + AFB (the unspecific findings are included) (see Table I). The number of false positive findings of the 216 biopsies taken from normal tissue is small (5 in WLB, 12 in AFB, 8 in AFB + WLB).

The positive predictive value indicates that most of the biopsies taken from suspicious areas were confirmed histologically, i.e. autofluorescence investigation caused an additional expense which was adequate to the final positive results. The high negative predictive value shows that only few

TABLE I Pilotstudy with 60 patients and 264 biopsies

| | Sensitivity | | PPV | NPV | Specificity |
|---|---|---|---|---|---|
| | Dysplasia/CIS | Tumors | Dysplasia CIS/Tumors | Dysplasia CIS/Tumors | |
| White light | 33% | 81% | 72% | 93% | 94% |
| White light + Autofluorescence | 83% | 83% | 63% | 96% | 89% |
| Relative factor | 2.80 | 1.03 | | | |

biopsies were false negatives which had been judged to be unspecific.

The specificity was very high even if autofluorescence and white light endoscopy were combined. We found the same amount of false positive biopsies characterizing inflamations or metaplasias (3 in WLB, 3 in AFB, 4 in AFB + WLB), scars or necrotic tissue (4 in AFB + WLB) in autofluorescence mode as well as in white light mode. In the combination WLB + AFB the amount of false positive results increased only slightly.

Figure 2 shows the middle lobe carina of a heavy smoker, which appears quite normal under WLB, while the fluorescence image shows a circumscript brown area, which indicates a histologically proven carcinoma *in situ*.

Figure 3 shows the left upper lobe carina of a patient who was investigated bronchoscopically because of a positive sputum cytology and negative X-ray image. White light bronchoscopy shows slight irregularity and fine roughening of the mucosa, which can be missed easily. Autofluorescence findings are characterized as marked brownish/bluish discoloration, indicating the transitional zone to the healthy tissue. Histologically we found microinvasive carcinoma within a larger area of carcinoma *in situ*.

Figure 4 shows the middle lobe bronchus of a heavy smoker who had undergone both lobectomy of left lower lobe and right upper lobe because of synchronous bronchial carcinomas. Years later we found a mild dysplasia by routine bronchoscopy in the dorsal wall of the middle lobe bronchus. The finding is marked only by a bluish color in the autofluorescence image. White light findings were normal.

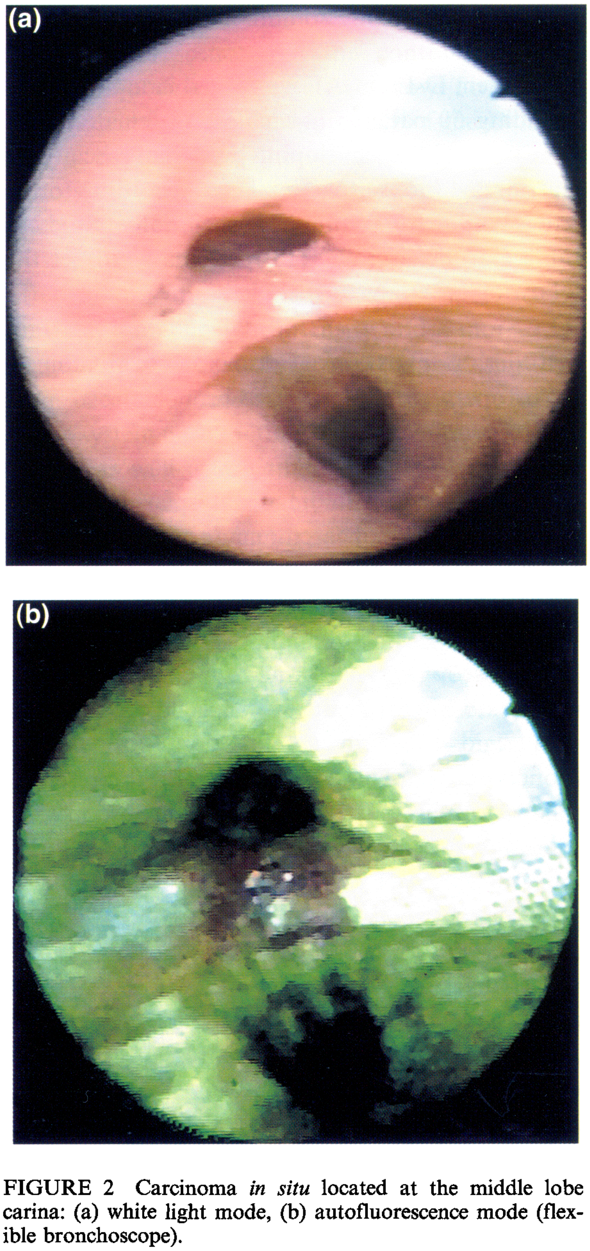

FIGURE 2 Carcinoma *in situ* located at the middle lobe carina: (a) white light mode, (b) autofluorescence mode (flexible bronchoscope).

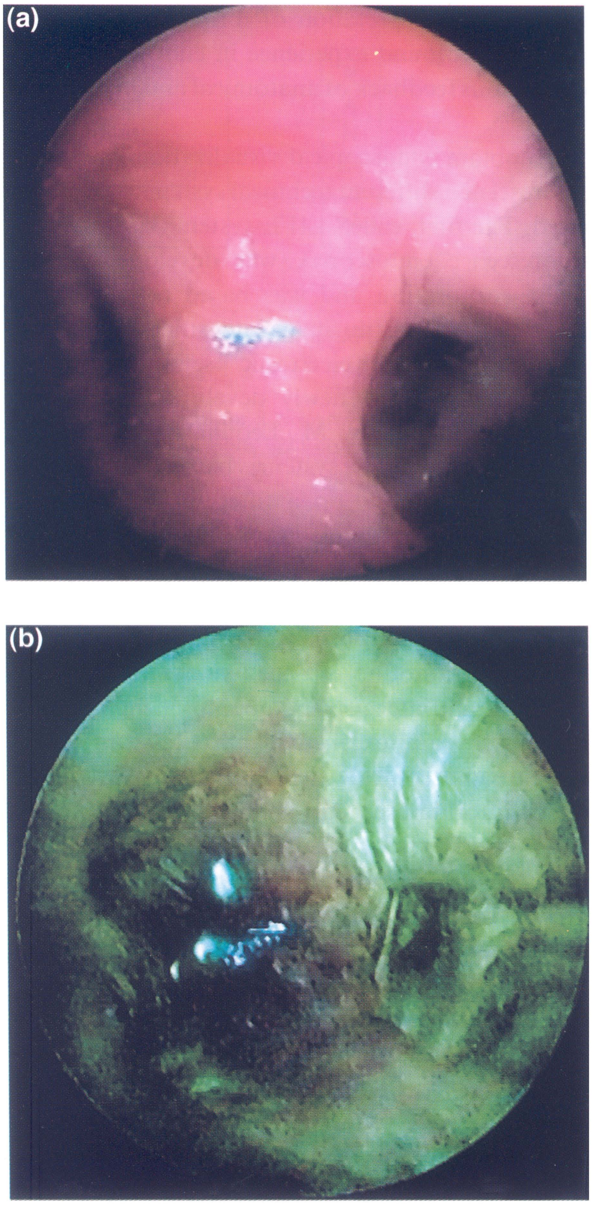

FIGURE 3 Microinvasive carcinoma *in situ* with a larger area of CIS: (a) white light mode, (b) autofluorescence mode (rigid bronchoscope).

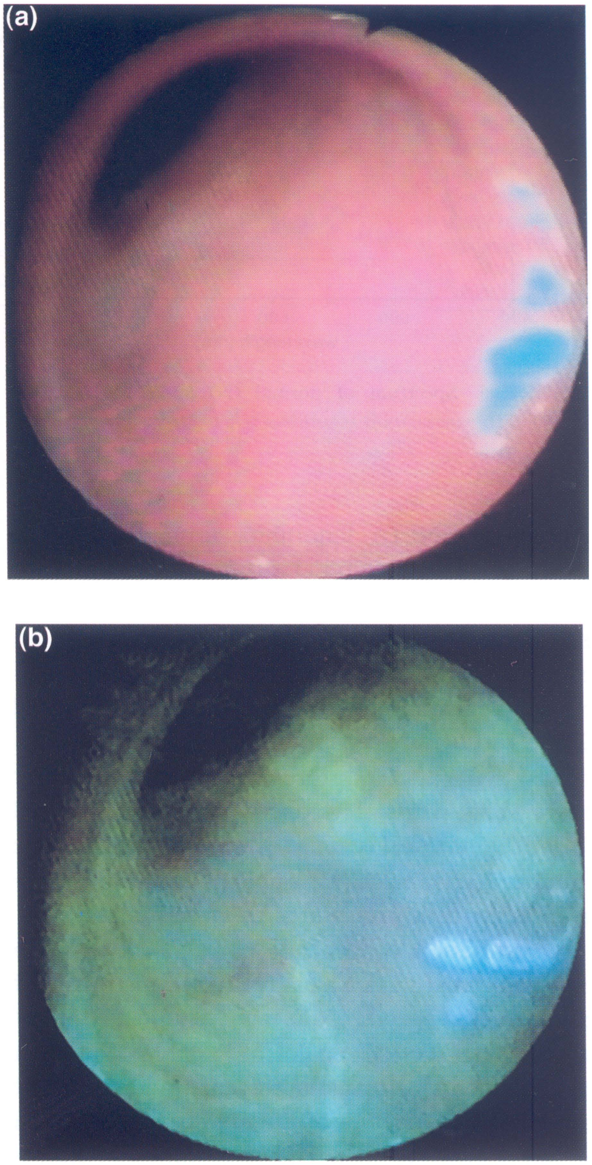

FIGURE 4 Mild dysplasia in the dorsal wall of the middle lobe bronchus: (a) white light mode, (b) autofluorescence mode (flexible bronchoscope).

Figure 5 shows an example of an autofluorescence spectrum taken from an area of severe dysplasia at the upper lobe carina and from normal tissue after excitation with blue light (380–460 nm). Both spectra are corrected to eliminate distance dependency [19]. Excitation of tumor tissue results in a strongly reduced intensity of the spectrum in the range between 500–600 nm. There is no difference between tumor and normal tissue for wavelengths above 650 nm [20].

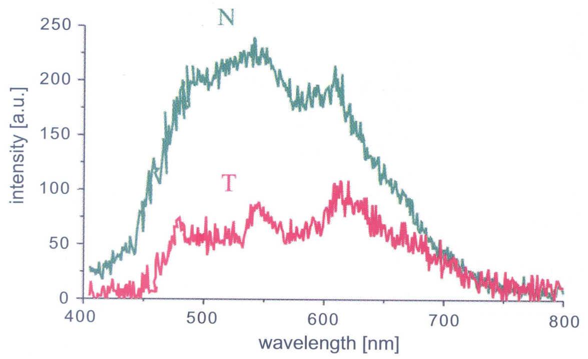

FIGURE 5 Spectrum of normal (N) and neoplastic (T) epithelium.

### DISCUSSION

The fluorescence system of the Karl Storz Company is based on the different fluorescence emissions of normal tissue and tumor tissue, either spontaneously as autofluorescence light or as drug induced fluorescence by selective accumulation of exogeneous applicated photosensitizers in the tumor tissue. This newly developed system can be used for both fluorescence diagnostic procedures. In the following only specific problems of autofluorescence diagnostic procedures are discussed.

The different concentrations of fluorophors in mucosal structures cause a characteristic image of color and a specific 3-D-image in the relief of mucosal surfaces. The color of the normal mucosa is dominated by a green fluorescence predominantly originating from connective tissue structures, which is interrupted by some darker, more brownish looking structures according to anatomy and morphology of smooth muscles within the mucosal membrane. A special 3-D-image is created by the tramline phenomenon of the pars membranacea. The normal mucosa of carinas is characterized by a homogeneous area of green color, while malignant tissue is indicated by circumscript areas of brownish or bluish discoloration within the green area of normal tissue. Other signs of malignancy are disturbance and/or dissolution of the normal anatomical structure and its specific color variations. The interpretation of different phenomena requires profound experience in endoscopy and a continuous feedback of anatomical and histological results.

To obtain endoscopical visualization with autofluorescence, different technical systems were developed. The first, called LIFE-system (laser induced fluorescence endoscopy), was developed by the Xillix corporation and was based on a helium–cadmium laser and an image intensifier camera. The result is an indirect electronic pseudo image. The components of the newly developed Karl Storz system, presented by M. Leonhard in detail are more convenient. The system is based on a modified xenon light source, on a filter in the ocular of the bronchoscope and an optional integrating camera which can be attached easily. The system allows the direct investigation by the naked eye and a quick and simple change between white light and autofluorescence mode by a footswitch. Light intensity and quality of the image of the system are excellent, and allow an exclusive bronchoscopical orientation and investigation under autofluorescence guidance.

All systems presented as part of this monograph aim at the early recognition of premalignant and/or early malignant changes in the bronchial mucosa. More than 50% of these changes in the central airways [4] are achievable with the flexible bronchoscope. The carcinogenesis lasts several years. Based on an investigation of section specimens of complete lungs Auerbach and Saccomano found a prevalence of 4.3% and 11.4% in slight and heavy smokers respectively [4,21,22]. Of all patients dying from bronchial carcinoma 15% had synchronous carcinomas. In patients with radiological occult bronchial carcinoma CIS was found in 20%, microinvasive carcinomas were found in 41% [4,5].

These favourable preconditions offer a chance of screening methods and endoscopic therapy.

To detect these premalignant and malignant changes various available autofluorescence systems were tested in studies. Up to now, the only large studies published have been about the Xillix system.

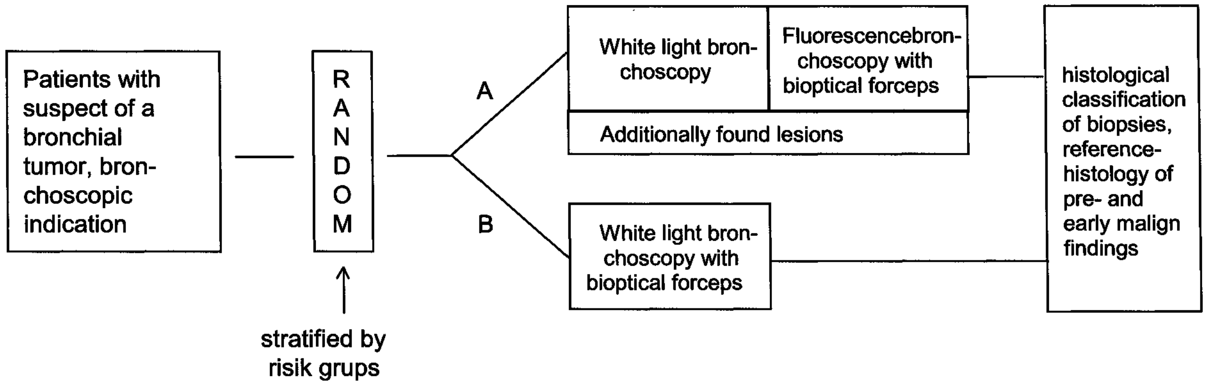

FIGURE 6 Concept for a two-arm multicenter study to evaluate the disgnostic effort of the additional fluorescence detection in contrast to a normal white light bronchoscopy.

In the British Columbia Cancer Agency Study the proportion of biopsy-proven moderate to severe dysplasia or CIS detected by WLB alone was 39%. With the addition of LIFE examination, the detection rate increased to 84% (WLB + AFB), whereas specificity decreased from 91% (WLB) to 86% (WLB + AFB) [23].

In the US multicenter study, sensitivity for dysplasia and carcinoma *in situ* increased from 9% by WLB to 57% by additional AFB (relative factor 6.3), specificity decreased from 90% to 66%. The prevalence of moderate to severe dysplasia and CIS was 14.6%, the overall prevalence of premalignant and malignant findings including invasive tumors was 20% [24].

In comparison to the above mentioned 4–11% [4,21,22] the prevalence of 15% for dysplasia and CIS published in the US study seems to be overestimated, especially considering the limitation of endoscopy to the central airways.

An explanation for the high prevalence reported could be that several biopsies were obtained from widespread malignant areas. In addition, some pathological findings might originate from the periphery of visible tumors. These topographic regions are not the areas of our interest. The primary aim of AFB is to detect isolated premalignant or malignant findings.

In comparison to the Canadian study the Karl Storz AF-system yielded a similar finding rate. The factor of 6.3 for additional AFB seems to be inadequatly high in the US multicenter study and is obviously caused by the low sensitivity for WLB of only 9%. The results of the published studies are at least problematic considering one further aspect: The study design did not definitely exclude a mutual influence in the endoscopical classification of the findings obtained by WLB or WLB + AFB.

We have designed a two-armed randomized multicenter study to evaluate AFB in general and our Karl Storz System in particular (Fig. 6)

One group of patients is investigated exclusively by white light and the second by a combination of WLB + AFB. To avoid mutual influence between the two methods it is planned to record biopsies in the direct periphery of tumors separately. So we hope to receive a realistic prevalence rate for isolated dysplasia and CIS. Finally the pathological findings should be related to biopsies and patients as well.

Following this procedure we expect to find a lower rate of premalignant and malignant findings, but we hope to receive a realistic evaluation of newly developed fluorescence devices for clinical use in early cancer detection.

***Acknowledgments***

We kindly thank our industrial partner Karl Storz GmbH & Co., Tuttlingen, Germany, for their support.

### *References*

[1] Baumgartner, R., Fisslinger, H., Jocham, D., Lenz, H., Ruprecht, L., Stepp, H. and Unsöld, E. A fluorescence imaging device for endoscopic detection of early stage cancer – instrumental and experimental studies, *Photochem. Photobiol.* 1987; **46**(5): 759–763.

[2] Stanzel, F., Häußinger, K., Pichler, J. and Sauer, W. The results of a pilot study with a new developed autofluorescence bronchoscopy system. 10th World Congress for Bronchology, June 1998, Budapest, contributed.

[3] Mueller, K.-M. and Gonzales, S. Präneoplasien und Frühkarzinom der Lunge – Histogenetische Aspekte des Bronchialkarzinoms. *Pneumologie* 1991; **45**: 971–976.

[4] Saccomano, G., Archer, V.E. and Auerbach, O. Development of carcinoma of the lung als reflected in exfollative cells. *Cancer* 1974; **33**: 256–270.

[5] Saccomano, G., Carcinoma *in situ* of the lung: Its development, detection, and treatment. *Semin. Respir. Med.* 1982; **4**: 156–160.

[6] Cortese, D.A., Pairolero, P.C., Bergstralh, E.J.*et al.* Roentgenopraphically occult lung cancer: A ten-year experience. *J. Thorac. Cardiovasc. Surg.* 1983; **86**: 373–380.

[7] Lam, S., Mac Auley, C., Le Riche, J.C., Ikeda, N. and Palcic, B. Early localization of bronchogenic carcinoma. *Diagnostic and Therapeutic Endoscopy* 1994; **1**: 75–78.

[8] Nasiell, M. Metaplasia and atypical metaplasia in the bronchial epithelium: A histopathologic and cytopathologic study. *Acta Cytol.* 1966; **10**: 421–427.

[9] Hayata, Y., Kato, H., Ono, J., Matsushima, Y., Hayashi, N., Saito, T. and Kawate, N., Fluorescence fiberoptic bronchoscopy in the diagnosis of early stage lung cancer. *Recent Results Cancer Res.* 1982; **82**: 121–130.

[10] Herly, L. Studies in selective differentiation of tissue by filtered ultraviolet light. *Cancer Research* 1943; 227–231.

[11] Sutro, C.J. and Burman, M.S. Examination of pathologic tissue by filtered ultraviolet radiation. *Arch. Pathol.* 1933; **16**: 346–349.

[12] Stanzel, F. and Häußinger, K. Bronchoskopische Fluoreszenzdiagnostik mit 5-Aminolävolinsaure (ALA). *Pneumologie* 1997; **5**: 250.

[13] Baumgartner, R., Jocham, D., Lenz, H. and Stepp, H., Unsöld-E Image-producing detection of porphyrin-marked tumors in the early stage. *Biomed. Tech. Berl.* 1989; **34** (Suppl): 24–25.

[14] Kennedy, J. and Pottier, R., Endogenous protoporphyrin IX, a clinically useful photosensitizer for photodynamic therapy. *J. Photochem. Photobiol.*, 1992; **14**: 275–292.

[15] Kriegmair, M., Stepp, H., Steinbach, P., Lumper, W., Ehsan, A., Stepp, H.G., Rick, K., Knüchel, R., Baumgartner, R. and Hofstetter, A., Fluorescence cystoscopy following intravesical instillation of 5-aminolevulinic acid: a new procedure with high sensitivity for detection of hardly visible urothelial neoplasias. *UROL.INT.* 1995; **55**(4): 190–196.

[16] Ischinger, T., Pesarini, A.C., Baumgartner, R., Coppenrath, K., Stepp, H. and Unsold, E. Spatial fluorescence imaging of atherosclerotic plaque: contrast enhancement by 2 wavelength laser stimulation, digital image processing and dye marking, *Z. Kardiol.* 1991; **80**(3): 207–214.

[17] Leunig, A., Rick, K., Stepp, H., Gutmann, R., Alwin, G., Baumgartner, R., and Feyh, J. Fluorescence imaging and spectroscopy of 5-aminolevulinic acid induced protoporphyrin IX for the dectection of neoplastic lesions in the oral cavity. Laryngo-Rhino-Otologie. 1996; **75**(8): 459–464.

[18] Battey, J.F., Brown, P.H., Gritz, E.R., Hong, W.K., Johnson, B.E., Karp, D.D., Mulshine, J.L., Shaw, G.L., Shopland, D.R., Sunday, M.E. and Szabo, E. Primary and secondary prevention of lung cancer an international association for the study of lung cancer workshop. *Lung Cancer* 1995; **12**: 91.

[19] Pichler, J.Stepp, H., Baumgartner, R., Häußinger, K., Brand, P. and Stanzel, F. Fluoreszenztumorlokalisation nach Applikation von 5-Aminolavolinsäure: Anwendung in der Pulmologie. Lasermedizin 1998; **13**:151.

[20] Hung, J., Lam, S. and LeRiche, J.C. and Palcic, B. Autofluorescence of normal and malignant bronchial tissue. *Lasers Surg. Med.*, 1991; **11**: 99.

[21] Auerbach, O., Petrick, T.G., Stout, A.P. *et al.* The anatomical approach to the study of smoking and bronchogenic carcinoma. *Cancer* 1956; **9**: 76–83.

[22] Lam, S. and Becker, H.D., Future Diagnostic procedures *Thoracic Endoscopy* 1996; **6**: 363–379.

[23] Lam, S. and MacAulay, C., Hung, J. and LeRiche, J. Detection of dysplasia and carcinoma *in situ* with a lung imaging fluorescence endoscope device, *The Journal of Thoracic and Cardiovascular Surgery* 1993; **105**(6).

[24] Lam, S., Kennedy, T., Unger, M. *et al.* Localizaton of bronchial intraephihelial neoplastic lesions by fluorescence bronchoscopy, *Chest* 1998; **3**: 696–702.

